# Mechanisms of Regenerative Potential Activation in Cardiac Mesenchymal Cells

**DOI:** 10.3390/biomedicines10061283

**Published:** 2022-05-31

**Authors:** Pavel M. Docshin, Andrei A. Karpov, Malik V. Mametov, Dmitry Y. Ivkin, Anna A. Kostareva, Anna B. Malashicheva

**Affiliations:** 1 Almazov National Medical Research Centre, Institute of Molecular Biology and Genetics, 197341 St. Petersburg, Russia; pdocshin@icloud.com (P.M.D.); kostareva_aa@almazovcentre.ru (A.A.K.); 2 Almazov National Medical Research Centre, Institute of Experimental Medicine, 194156 St. Petersburg, Russia; karpov_aa@almazovcentre.ru; 3 Center of Experimental Pharmacology, Saint Petersburg State Chemical Pharmaceutical University, 197022 St. Petersburg, Russia; dmitry.ivkin@pharminnotech.com; 4 Department of Pathophysiology, Pavlov First Saint Petersburg State Medical University, 197022 St. Petersburg, Russia; m_mametov@inbox.ru; 5 Laboratory of Regenerative Biomedicine, Institute of Cytology, Russian Academy of Science, 194064 St. Petersburg, Russia

**Keywords:** myocardial infarction, heart regeneration, cardiac mesenchymal cells, Notch signaling pathway, early remodeling of the heart

## Abstract

Recovery of the contractile function of the heart and the regeneration of the myocardium after ischemic injury are contemporary issues in regenerative medicine and cell biology. This study aimed to analyze early transcriptional events in cardiac tissue after infarction and to explore the cell population that can be isolated from myocardial tissue. We induced myocardial infarction in Wistar rats by permanent ligation of the left coronary artery and showed a change in the expression pattern of Notch-associated genes and *Bmp2/Runx2* in post-MI tissues using RNA sequencing and RT-PCR. We obtained primary cardiac mesenchymal cell (CMC) cultures from postinfarction myocardium by enzymatic dissociation of tissues, which retained part of the activation stimulus and had a pronounced proliferative potential, assessed using a “xCELLigence” real-time system. Hypoxia in vitro also causes healthy CMCs to overexpress Notch-associated genes and *Bmp2/Runx2*. Exogenous activation of the Notch signaling pathway by lentiviral transduction of healthy CMCs resulted in a dose-dependent activation of the *Runx2* transcription factor but did not affect the activity of the *Bmp2* factor. Thus, the results of this study showed that acute hypoxic stress could cause short-term activation of the embryonic signaling pathways Notch and Bmp in CMCs, and this interaction is closely related to the processes of early myocardial remodeling after a heart attack. The ability to correctly modulate and control the corresponding signals in the heart can help increase the regenerative capacity of the myocardium before the formation of fibrotic conditions.

## 1. Introduction

Myocardial infarction remains one of the major medical problems of a non-infectious character that kills and disables the world’s population [1]. The search for an effective therapeutic strategy is still ongoing, and many studies are aimed at exploring the possibilities of myocardial cell therapy, but the cellular mechanisms of post-infarction cardiac recovery are still unclear. According to traditional concepts, the human heart is a definitively differentiated organ; however, foci of cell proliferative activity may occur in the heart, the intensity of which increases in the peri-infarct zone [2]. The discovery of cardiac stem cells stimulated the development of new approaches in myocardial cell therapy [3], but the ability of these cells to directly generate cardiomyocytes remains controversial.

The turnover of contractile cardiomyocytes in a healthy myocardium is approximately 0.5–2% per year [4,5], and these numbers slightly increase with heart damage [2]. The renewal of cardiomyocytes occurs mainly due to the re-entry of cells into the cell cycle, but not due to the differentiation of the cardiac stem cells themselves [5]. Mesenchymal stem cells (including cardiac stem cells) possibly participate in regenerative processes in the myocardium, releasing paracrine factors that provide myocardial protection, neovascularization, remodeling, and differentiation of the heart [6].

There is evidence that the mechanisms of myocardial functional recovery are contained in paracrine intercellular signaling, and their activation can occur precisely during the early response to damage. In this regard, the study of the mechanisms of early activation of regenerative processes in post-infarction tissue is an urgent issue. Although the existence of resident cardiac progenitor cells has been questioned [5], it is clear that myocardial regeneration exists, but the mechanisms remain undefined. This study aimed to analyze early transcriptional events in cardiac tissue after infarction and to explore the cell population that can be isolated from myocardial tissue.

Here we report that acute hypoxic stress affects the activation of the Notch signaling pathway and *Bmp2/Runx2* genes in cardiac mesenchymal cells, and the action of these pathways is associated with early myocardial remodeling processes. In this study, we induced myocardial infarction in rats and, 8/24 h after surgery, we isolated post-infarction tissues and primary CMC cultures with a pronounced proliferative potential. We showed a change in the expression pattern of Notch-associated genes and *Bmp2/Runx2* in postinfarction tissues and CMCs using RNA sequencing and RT-PCR. Hypoxia in vitro also causes healthy CMCs derived from sham-operated rats to upregulate Notch-associated genes and *Bmp2/Runx2*. Exogenous activation of the Notch signaling pathway led to dose-dependent activation of the transcription factor Runx2 but did not affect the activity of the Bmp2 factor.

## 2. Materials and Methods

### 2.1. Ethics Statements and Animals

We obtained permission from the local ethics committee of the Almazov National Medical Research Centre for conducting animal experiments. Male Wistar rats (Pushchino, Russia) of the same age and weight between 200 and 250 g were used in the experiment. Animals were kept in separate plastic cages with free access to water and standard diet food during the experiment. All experiments were performed following the Guide for the Care and Use of Laboratory Animals.

### 2.2. Induction of Myocardial Infarction In Vivo

Male Wistar rats (*n* = 16) were anesthetized with chloral hydrate (2 mg/kg intraperitoneally), intubated, and vented (SAR-830P; CWE, Inc., Ardmore, PA, USA) using room air with a tidal volume of 2 mL/100 g and a frequency of 60 breaths per minute. The core body temperature was maintained at 37.0 ± 0.5 °C using a feedback heating pad (TCAT-2LV controller; Physitemp Instruments Inc., Clifton, NJ, USA). Registration of heart rate and arrhythmias was monitored by electrocardiography. After thoracotomy, the heart was visualized through the fourth intercostal space. Further, in a blunt way, using the branches of anatomical tweezers, the pericardium was removed. At the border of the free edge of the left atrial appendage, the left coronary artery (LCA) was visualized, under which a ligature (prolene 6/0, Ethicon, Germany) was applied, directly at the edge of the left atrial appendage [7]. Myocardial ischemia was confirmed by visual examination of the anterior surface of the heart and the elevation of the ST segment on the ECG.

### 2.3. RNA-seq Library Preparation

We excised the post-infarction area, including the peri-infarction zone (the area was slightly visible and had a whitish hue, and was under the ligature), from the left ventricle of the ischemic heart to obtain post-infarction tissues and isolation of the primary CMC cell culture. We isolated total RNA according to the manufacturer’s protocol (Eurogen, Russia) from postinfarction tissues and CMCs (*n* = 3); RNA obtained from sham-operated rats from healthy myocardial tissues and CMCs (*n* = 3) was used as a control. The quality and quantity of the isolated RNA were checked on a NanoDrop 1000 spectrophotometer (Thermo Fisher Scientific) and an Agilent 2100 bioanalyzer (Agilent Technologies). A total of 1 μg of total RNA was used to create libraries using the TruSeq RNA sample preparation kit (Illumina) following the low sample (LS) protocol from the manufacturer’s instructions.

### 2.4. Differential Gene Expression Analysis

Raw RNASeq reads were aligned with STAR 2.7 against Rnor_6.0 (GCA_000001895.4) and transcript annotations (ensemble 97) [8]. Differential expression analysis was performed using the DESeq2 Bioconductor package [9]. Genes with a p-value of 0.05 or less were called differentially expressed genes. Comparisons were made between ischemic tissues/cells and control tissues/cells. Data analysis and visualization (PCA Plot and Volcano Plot) were performed using Phantasus (version: 1.7.3, build: master-709) [10].

### 2.5. Ingenuity Pathway Analysis

Bioinformatic analysis was performed using ingenuity pathway analysis (IPA; Qiagen Silicon Valley, Redwood City, California, USA. Available online: http://www.ingenuity.com (accessed on 24 September 2020)) of differentially expressed genes to determine the interactions of genes and related networks using the default Rattus norvegicus background for settings. All the genes that passed the significance filter were identified as focus genes and uploaded to the IPA for further functional and network analysis. The specificity of connections within the network for each focus gene was calculated by the percentage of its connections with other significant genes in the database. Canonical pathway analysis identifies the paths from the IPA library that were most significant for the input data set.

### 2.6. Isolation of Cardiac Mesenchymal Cells

The heart was taken 8 and 24 h after surgery. Cardiac mesenchymal cells were obtained from the ischemic myocardial zone by grinding a tissue fragment followed by enzymatic treatment with type 2 collagenase (Worthington) solution (2 mg/mL) for 90 min in an incubator (37 °C, 5% CO^2^, humidity 99%); a healthy myocardium obtained from rats 24 h after the sham operation was used as a negative control [11]. The cell suspension was centrifuged at 300 x *g* for 5 min, and cells were resuspended in growth medium (two times), seeded on the flask, and cultured in an incubator (37 °C, 5% CO^2^, and 99% humidity). Medium for cardiac mesenchymal cells contains: 70% DMEM/F_12_ (Invitrogen, Waltham, MA, USA), 20% ECM (Invitrogen, USA), 10% fetal bovine serum (HyClone, Logan, UT, USA), 100 μM MEM NEAA amino acid solution (Gibco, Grand Island, New York, USA), 2 mm L-Glutamine (Gibco, USA), a mixture of penicillin (100 u/mL) and streptomycin (100 μg/mL) (Gibco, USA). In the next three days, we replaced it with a fresh culture medium once per day. On the third day, we removed large tissue debris and then continued to cultivate until the confluent state (~1 week). In the obtaining culture, live cardiomyocytes were absent, and the cell population was homogeneous. In this study, we used cells derived from rats 24 h after surgery, between 1 and 3 passages.

### 2.7. Assessment of Proliferative Activity of Cardiac Mesenchymal Cells

Cell proliferation was monitored in real-time using the xCELLigence RTCA DP Real-Time Cell Analyzer system. We used impedance as an indication recorded by the xCELLigence system to evaluate cell proliferative ability [12]. The system measures electrical impedance through oncoming microelectrodes embedded in the bottom of the electronic plates. The impedance measurement, which is displayed as a cell index (CI) value, provides quantitative information about the biological status of the cells, including the number of cells and their viability. Five thousand cells were sown in each well of the E-Plate (in 100 μL of cell suspension). The impedance value of each well was automatically monitored by the xCELLigence system for 72 h and expressed as the CI value. The obtained data were processed in the RTCA Software program (version number 1.0.0.1304).

### 2.8. In Vitro Hypoxia Induction

We used cardiac mesenchymal cells from sham-operated rats. The cells were seeded on 5 cm Petri dishes and cultured in the medium for the CMCs. The next day we transferred the cells into an incubator, where it is possible to adjust the level of oxygen in the chamber. Cardiac mesenchymal cells were hypoxic for 8 and 24 h with oxygen levels of 1% and 5% (37 °C, 5% CO^2^, and 99% humidity); as a control, we used cells under normoxic conditions (37 °C, 5% CO^2^, 20% O^2^, and 99% humidity).

### 2.9. Real-Time PCR

RNA was isolated from myocardial tissues and primary cell cultures using a Trisol analog called ExtractRNA (Eurogen, Russia). Reverse transcription was performed using MMLV reverse transcriptase and the MMLV RT kit (Eurogen, Russia) according to the recommendations of the manufacturer. All samples were pretreated with DNase. We used a 5-fold reaction mixture qPCR mix-HS SYBR with intercalating dye SYBR Green I (Eurogen, Russia) for real-time PCR. cDNA (50 ng), forward and reverse primers (10 μM each) were added to the mixture, and the final volume was adjusted with sterile water to 25 μL. The sequences of the primers used: *Gapdh* (F: CCAGTATGACTCTACCCACG, R: CATTTGATGTTAGCGGGATCTC), *Notch1* (F: CAATGAGTGTGACTCACGGC, R: GCACAAGGTTCTGGCAGTTG), *Notch2* (F: CCGTGGGGCTGAAGAATCTC, R: CTTTCTTTGGCTGGGGTCCT), *Notch3* (F: GCCTAGTCCAGCAACTGCTAC, R: GGGAACAGATATGGGGTGTGG), *Dll1* (F: TAACCCCGATGGAGGCTACA, R: GCACCGTTAGAACAAGGGGA), *Dll4* (F: GCAGCTGTAAGGACCATGAGA, R: TTCACAGTGCTGGCCATAGT), *Jag1* (F: CGCCCAATGCTACAATCGTG, R: TCTTGCCCTCGTAGTCCTCA), *Hes1* (F: ACCAAAGACAGCCTCTGAGC, R: TTGGAATGCCGGGAGCTATC), *Runx2* (F: TCCCTCCGAGACCCTAAGAAA, R: GCTGCTCCCTTCTGAACCTAC), *Bmp2* (F: CTGCCATGGGGAATGTCCTT, R: TGCACTATGGCATGGTTGGT), *Hif-1a* (F: GGCGAGAACGAGAAGAAAAATAGG, R: AGATGGGAGCTCACGTTGTG), *Vegf-a* (F: GCAGCGACAAGGCAGACTAT, R: TGGCACGATTTAAGAGGGGA), *Ccnd1* (F: CTTACTTCAAGTGCGTGCAGAG, R: TTCATCTTAGAGGCCACGAACA), *Hes7* (F: CATCAACCGCAGCCTAGAAGAG, R: CACGGCGAACTCCAGTATCTCT), *Hey1* (F: CCTGGCTATGGACTATCGGAG, R: AGGCATCGAGTCCTTCAATGAT), *Myc* (F: CAGCTCGCCCAAATCCTGTA, R: TGATGGGGATGACCCTGACT). The polymerase chain reaction was carried out using a 7500 Real-Time PCR System (7500 Software v2.3, Life Technologies Ltd, Paisley, UK). Quantitative PCR was performed for 40 cycles. Data analysis was conducted using the 2^-ΔΔCT^ method; relative gene expression was normalized on the GAPDH housekeeping gene.

### 2.10. Statistical Analysis

Analysis of the data was performed using Microsoft Excel and GraphPad Prism Software (version 9.3.1(350)). The significance of differences between the groups was evaluated using the unpaired nonparametric Mann–Whitney test. The differences were considered significant at *p* < 0.05. All experiments were repeated three times.

## 3. Results

### 3.1. The Gene Expression Profile Changes Significantly in Myocardial Tissues 24 h after Infarction

**Figure 1:**
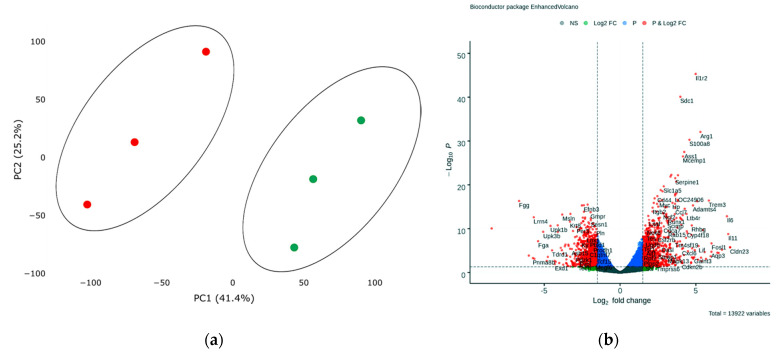
(**a**) Principal component analysis (PCA) displays variability in gene expression in healthy (green dots, *n* = 3) and postinfarction (orange dots, *n* = 3) myocardial tissues using the Phantasus Web Tool. Major component 1 (PC1) on the *x*-axis and PC2 on the *y*-axis accounted for 41.4% and 25.2% of the total variability in gene expression, respectively. PC1 covers a significant part (41.4%) of the experimental variability and largely reflects the difference between the two states of the heart muscle. PC2 represents experimental variability (25.2%) associated with the difference in gene profile between various biological repeats (laboratory animals). The samples are visually divided into two main groups. (**b**) Volcano plots showing differentially expressed genes (adjusted *p*-value < 0.05) in post-infarction samples compared to controls, performed using Bioconductor software in R. The *y*-axis corresponds to the mean expression value of −log10 (*p*-value), and the *x*-axis displays the log2 (fold-change) value. The red dots represent significantly differentially expressed genes.

In order to assess transcriptional changes in the heart during acute hypoxic stress, we induced myocardial infarction in Wistar rats by permanent ligation of the left coronary artery. Twenty-four hours after the operation, we took the postinfarction area of the myocardium, including the periinfarction zone, for subsequent isolation of total RNA and preparation of libraries. As a control, we used a healthy area of the myocardium obtained from sham-operated rats. We analyzed the genetic profile of postinfarction tissues by RNA sequencing. PCA showed significant variability in data for the first and second components (Figure 1a). The transcriptional profiles of post-infarction and healthy tissues were divided and formed separate clusters. Analysis of differentially expressed genes (DEGs) showed that in postinfarction tissues, 1241 genes were upregulated and 1256 downregulated (adjusted *p*-value < 0.05) (Figure 1b). The top 50 DEGs include genes that determine the epithelial–mesenchymal transition, as in wound healing and fibrosis (*CD44, GPC1, SDC1, VCAN, PLAUR, SERPINE1, PMEPA1, PVR*); genes encoding proteins involved in glycolysis and gluconeogenesis (*ANGPTL4, SLC16A3*); genes upregulated by STAT5 in response to IL2 stimulation (*MYC, SLC1A5, IL1R2, SELP*), as well as genes that are regulated by NF-kB in response to TNF, in response to low oxygen levels (hypoxia), and determine the inflammatory response (Table S1).

### 3.2. Early Remodeling Processes are Enhanced in the Postinfarction Myocardium, which is Involved Components of the BMP and NOTCH Signaling Pathways

To assess the effect of acute hypoxic injury on dysregulation of canonical signaling pathways and biological functions in cardiac tissue 24 h after the onset of infarction, we performed GSEA and canonical pathway analysis of DEGs using Qiagen software. DEGs were uploaded into Qiagen Ingenuity Pathway Analysis (IPA) to identify enriched pathways in the dataset, after filtering for significance (adj. *p* < 0.05). In total, about 152 canonical signaling and metabolic pathways were identified, the significance of which was higher than −log (P)> 1.3, and only 82 signaling pathways had absolute z-scores more than 1.0 (Figure S1A).

In the heart after myocardial infarction, massive cell death occurs in the affected area and a sustained formation of an inflammatory response, in particular, aimed at providing reparative processes associated with early myocardial remodeling [13]. We observed a shift in the pattern of gene expression towards early myocardial remodeling 24 h after the induction of acute infarction. According to this, an affective *‘Apelin Cardiac Fibroblast Signaling Pathway’* (ratio—0.455; z-score—−2.333; *p*-value—5.67× 10^−4^), which means the activation of cardiac fibroblasts and their differentiation into myofibroblasts and causes the formation of cardiac fibrosis, leading to heart failure, and *‘Remodeling of Epithelial Adherents Junctions’* (ratio—0.382; z-score—2.449; *p*-value—1.71 × 10^−5^), *‘Inhibition of Matrix Metalloproteases’* (ratio—0.344; z-score—−1.265; *p*-value—4.57× 10^−3^), or *‘TGF-β Signaling’* (ratio—0.218; z-score—1.5; *p*-value—4.86× 10^−2^) were found in dataset (Figure S1A). Additionally, signaling pathways involved in the regulation of the cell cycle and proliferative activity (‘Cell Cycle Control of Chromosomal Replication’, ‘PI3K/AKT Signaling’, ‘Aryl Hydrocarbon Receptor Signaling’, ‘Cell Cycle Regulation by BTG Family Proteins’, ‘STAT3 Pathway’, ‘HIF1α Signaling’, ‘Cyclins and Cell Cycle Regulation’) and migration activity (‘Regulation of Actin-based Motility by Rho’, ‘Actin Cytoskeleton Signaling’) were activated in postinfarction tissues. 

Gene networks generated using the IPA program, which reflect forms of non-canonical signaling, were noted for various upregulated and downregulated genes involved in the growth and development of tissues and cells (Figure S1B). More than 100 molecules have been identified by gene-set enrichment analysis that are involved in the formation of the immune response, the growth and development of the cardiovascular system, the processes of cell division, and changes in cell morphology are affected. The main bio-functions and diseases are listed (Table S2). It should be noted that proliferative processes, including smooth muscle cells, differentiation of connective tissue cells, and organization of sarcomere are activated in the ischemic heart (Figure S2).

In these processes, the activity of *Bmp2* from the TGF-b subfamily and several components of the Notch signaling pathway was noted, such as the *Notch2* receptor, the target genes *Myc, Ccnd1*, and the *Runx2* transcription factor [14,15], which were upregulated, and the target gene *Hey1*, which was downregulated. In particular, *Bmp2* from the TGF-b subfamily was identified among the key regulators. A complete list of overexpressed components of the Notch signaling pathway is presented (Table S3).

### 3.3. Activation of Notch Signaling Pathway Components and Bmp2/Runx2 in Post-Infarction Myocardial Tissues

In order to confirm the RNA sequencing data that *Bmp2* and components of the Notch signaling pathway are activated in post-infarction tissues, we analyzed them using qPCR. Additionally, we took into account an even earlier time point after the induction of myocardial infarction in rats, 8 h, to assess the difference in gene expression between the two time intervals.

**Figure 2:**
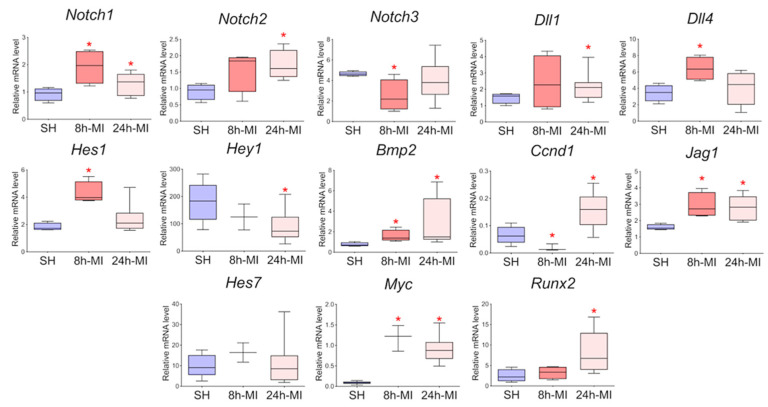
The dynamics of the expression of the Notch signaling pathway and *Bmp2/Runx2* in tissues by quantitative PCR analysis. SH—healthy myocardium from sham-operated rats (*n* = 3); 8 h-MI—tissue from the post-infarction zone of the myocardium from rats 8 h after induction (*n* = 4); 24 h-MI—tissue from the post-infarction zone of the myocardium from rats 24 h after induction (*n* = 9). Vertical—the relative amount of mRNA in each group, measured by the 2^−ΔΔCT^ method; box plots with whiskers at min to the max are presented. * The asterisk shows significant differences between SH and MI groups at *p* < 0.05 (unpaired nonparametric Mann–Whitney test).

We showed (Figure 2) that acute hypoxia in vivo activates the expression of Notch signaling pathway components and Bmp2 in the ischemic myocardium compared to a healthy heart. The obtained qPCR data were in accordance with the RNA sequencing results for the 24 h point.

### 3.4. Postinfarction Cardiac Mesenchymal Cells had a Pronounced Ability to Proliferate

**Figure 3:**
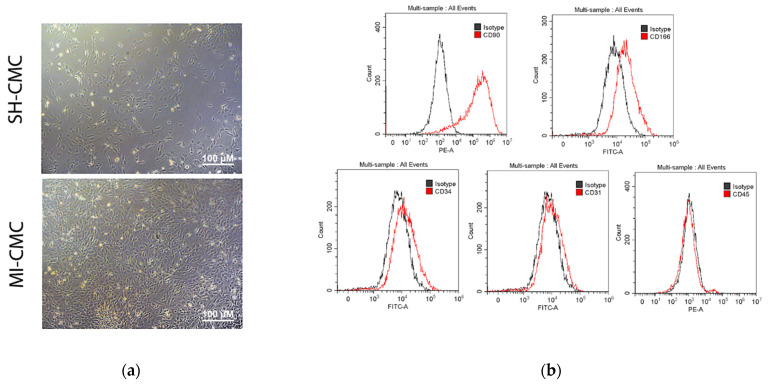
(**a**) Cardiac mesenchymal cells (CMCs) derived from healthy (SH-CMC) and postinfarct (MI-CMC) myocardium of rat during 5-day cultivation. CMCs were isolated 24 h after myocardial infarction. SH-CMC were obtained from rat hearts after sham operation. The primary cell culture of CMCs demonstrated the ability to form fibroblast colony-forming units (CFU-F) and had adhesion to a culture plastic. (**b**) Immunophenotyping of primary cultures of CMC using flow cytometry. The histograms show the expression levels of surface markers CD31, CD34, C45, CD90, and CD166 (red lines) concerning isotype (gray lines).

We induced myocardial infarction in Wistar rats to obtain primary cultures of cardiac mesenchymal cells from the postinfarction area (including the periinfarction zone) of the myocardium 8 and 24 h after surgery; cardiac mesenchymal cells of the ventricular myocardium of the healthy heart of sham-operated rats were used as a negative control (Figure 3a). The phenotypic characterization of this cell type is complex, and there is no specific marker or combination of markers for identifying mesenchymal stem cells (MSCs) [16,17]. The International Society for Cellular Therapy has established the following criteria for the identification of MSCs: adhesion to plastic, expression of markers, the adipogenic and osteogenic differentiation ability, and the ability to form fibroblast colony-forming units (CFU-F) [17,18]. Here, we use the term cardiac mesenchymal cells (CMC) because of some terminological differences associated with the correct classification of these cells [19,20]. The obtained CMC primary cultures clearly expressed CD90 and were positive for CD166, which was previously described as a marker of cardiac mesenchymal cells and one of the populations of cardiac stem cells obtained from cardiospheres [21]. They were also negative for endothelial markers and did not belong to hematopoietic markers, namely negative for CD45, CD31, and CD34 [17] (Figure 3b).

**Figure 4:**
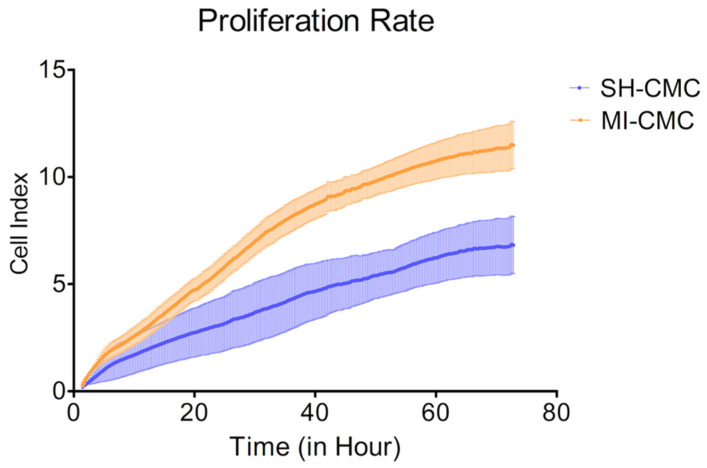
The proliferation rate of postinfarction cardiac mesenchymal cells (MI-CMC, orange curve, *n* = 9) and healthy cardiac mesenchymal cells (SH-CMC, blue curve, *n* = 3). Each curve represents the average values between the samples and the standard error of the mean (vertical lines). Horizontal—the time during which the measurements of proliferative activity were taken every 15 min. The experiment lasted for 72 h. Vertical—the value of the cell index, which reflects the quantitative information about the biological status of cells, including their number and viability. The seeding density was 5000 cells per well. The significance of differences between the SH-CMC and the MI-CMC is *p* < 0.05 with D’Agostino and Pearson omnibus normality test.

To evaluate and compare the proliferative activity of postinfarction and healthy cardiac mesenchymal cells obtained one day after surgical procedures, we used the xCELLigence system to monitor cell proliferation in real-time. We found (Figure 4) that postinfarction CMCs (orange curve) have a more pronounced potential for proliferation and, accordingly, have an activation stimulus in response to acute hypoxic stress than healthy myocardial cells (blue curve). The experiment lasted for 72 h with a frequency of measurements every 15 min.

### 3.5. The Gene Expression Profile of Postinfarction CMC is Altered

**Figure 5:**
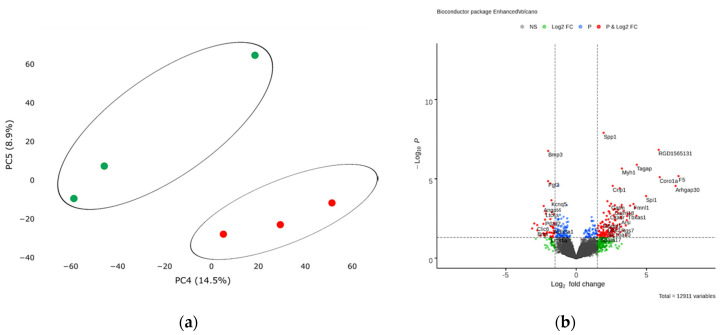
(**a**) Principal component analysis (PCA) displays variability in gene expression in healthy (green dots, *n* = 3) and postinfarction (orange dots, *n* = 3) CMCs using the Phantasus Web Tool. Major component 1 (PC4) on the *x*-axis and PC5 on the *y*-axis accounted for 14.5% and 8.9% of the total variability in gene expression, respectively. The samples are visually divided into two main groups. (**b**) Volcano plots showing differentially expressed genes (*p*-value < 0.05) in post-infarction samples compared to controls, performed using Bioconductor software in R. The *y*-axis corresponds to the mean expression value of -log10 (*p*-value), and the *x*-axis displays the log2 (fold-change) value. The red dots represent significantly differentially expressed genes.

Next, we analyzed transcriptomic profile of cardiac mesenchymal cells from the postinfarction area and from healthy hearts of sham-operated rats by RNA sequencing. Principal component analysis showed the main patterns in the resulting dataset. PC4 covers an insignificant part (14.5%, *p* < 0.05) of the experimental variability and reflects a relatively small difference between the two CMC states, which may indicate a gradual change in the expression profile during cell culture under normal conditions (Figure 5a).

Analysis of RNA sequencing data revealed 13 differentially expressed genes (adjusted *p*-value <0.05) in the obtained post-infarction cell cultures. Among the activated genes, the expression of *Spp1, RGD1565131, Tagap, Myh1* can be noted, while the expression of *Bmp3, Fgl2, Sfrp4,* on the contrary, was reduced in postinfarction cardiac mesenchymal cells (log2 (fold-change) ≥ 1.9) (Figure 5b). A complete list of differentially expressed genes (DEGs), sorted by adjusted p-value, is presented (Table S4). We collected the 50 top highly expressed genes, sorted by statistical criteria (Table S5).

We found that the expression of *Twist1* and the target genes Notch *Hey2* and *Ccnd1* are reduced in postinfarction cardiac mesenchymal cells. In addition, the activity of the *Thbs2* gene, which may be responsible for the inhibition of angiogenesis, and *Dnm1*, which is involved in vesicular transport, decreased (*p*-value> 0.05) (Table S6).

### 3.6. GSE Analysis and Canonical Pathway Analysis of DEGs Showed that the Pattern of Gene Expression Characteristic of the Ischemic Heart is Partially Retained in MI-CMC

**Figure 6:**
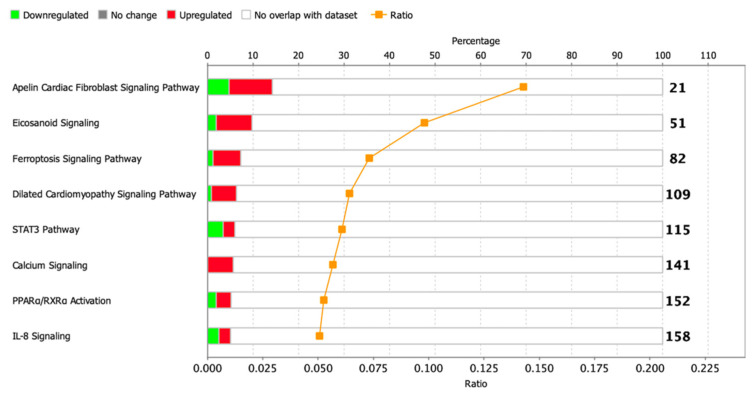
Dysregulated signaling pathways in postinfarction CMC samples identified by IPA analysis. The histogram represents the dysregulated canonical signaling pathways in a stacked bar chart by Fisher’s Exact Test p-value (*p*-value > 0.05, z-score > 1). The ratio plot shows the number of significant genes expressed in the data versus the total number of genes in that particular signaling pathway.

To identify enriched signaling pathways in the dataset, genes were loaded into Qiagen Ingenuity Pathway Analysis (IPA) after significance filtering (*p*-value < 0.05). In total, about 14 canonical signaling pathways were identified, including Metabolic Pathways and Signaling Pathways, the significance of which was greater than −log (P)> 1.3, and only 1 STAT3 signaling pathway had absolute z-scores more than 2.0 (Figure 6). A complete list of this data is provided in Table S7.

We conducted a core analysis in the IPA program to find key diseases and bio-functions in the CMCs, and we identified about 12 categories in which processes related to diseases or functions were affected (*p*-value—0.05), and only in 3 of them, the z-score was more than 2. It was interesting to note that a partial pattern of genes associated with heart failure persisted in post-infarction cells (*‘Failure of heart’*, *p*-value = 8.25× 10^−3^, z-score = 2.177), and along with this, the modulation of processes associated with neovascularization and cell migration was reflected. At the same time, the *‘Expansion of cells’* parameter was suppressed (*p*-value = 6.09× 10^−3^, z-score = −2.219), which may be the result of a delay in the cell cycle, since DNA synthesis was also slightly increased (p-value = 1.31× 10^−2^, z-score = 1.077). A complete list of this data is provided (Table S8).

We constructed probabilistic networks of dysregulated genes using the IPA program based on the detected activated/repressed genes and those that were statistically filtered but present in the data set (Table S9). We combined probabilistic networks based on related processes and key regulators and found that one of the Notch *Hey2* targets is reduced in MI-CMCs.

### 3.7. The Expression of the Jag1 and Hes1 Genes of the Notch Signaling Pathway and Bmp2/Runx2 Factors is Preserved in Cell Culture

**Figure 7:**
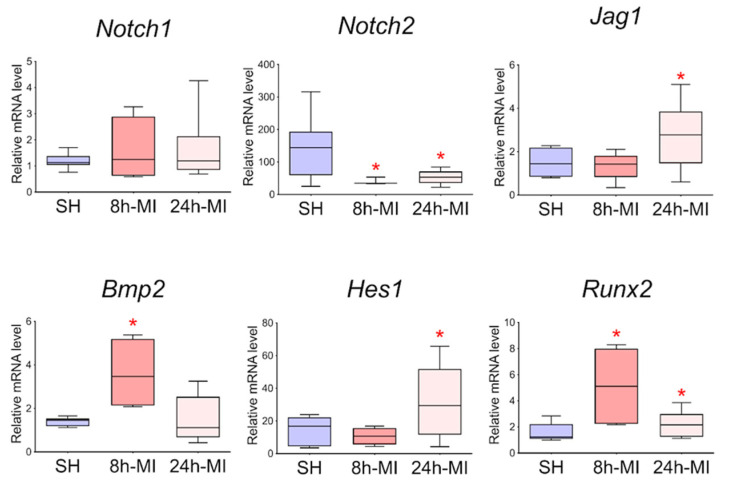
Dynamics of expression of Notch signaling pathway components and *Bmp2/Runx2* genes in cardiac mesenchymal cells using quantitative PCR analysis. SH—cells from a healthy myocardium of sham-operated rats (*n* = 3); 8 h-MI—post-infarction cells from rats 8 h after induction (*n* = 4); 24 h-MI—post-infarction cells from rats 24 h after induction (*n* = 9). Vertical—the relative amount of the mRNA in each group, measured by the 2^-ΔΔCT^ method; box plots with whiskers at min to the max are presented. The asterisk shows significant differences between the SH and MI groups at *p* < 0.05 (unpaired nonparametric Mann–Whitney test).

In order to approve the results of RNA sequencing and evaluate the level of expression of Notch signaling components using real-time PCR with reverse transcription, passages 1–3 cells were obtained from rat tissues 8 and 24 h after induction of myocardial infarction. In particular, we assessed the level of expression of *Bmp2* and *Runx2*, the dysregulation of which was noted in the transcriptome. Since the BMP signaling pathway plays an essential role in cardiogenesis, as does the Notch signaling pathway, and *Runx2* can be a target gene for both types of signaling and has also been identified in cardiac development, according to our hypothesis, *Bmp2* and *Runx2* can be early remodeling genes. We found (Figure 7) that in postinfarction cardiac mesenchymal cells, the activation potential is partially preserved, which is expressed in increased expression of the *Bmp2* and *Runx2* genes, and Jag1 and *Hes1* genes of the Notch signaling pathway compared to cells obtained from a healthy heart.

### 3.8. Activation of Notch Signaling Pathway Components and Bmp2/Runx2 Factors in Cardiac Mesenchymal Cells during In Vitro Hypoxia Induction

**Figure 8:**
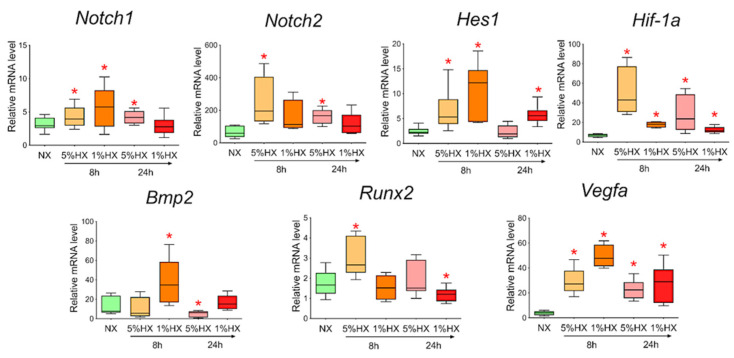
Dynamics of expression of Notch signaling pathway components, *Bmp2/Runx2* factors, and hypoxic stress markers in cardiac mesenchymal cells obtained from healthy myocardium of sham-operated rats by quantitative PCR analysis. NX—healthy cells in a state of normoxia (*n* = 3); 5% HX—healthy cells in a state of hypoxia with an oxygen level of 5% (*n* = 3); 1% HX—healthy cells in a state of hypoxia with an oxygen level of 1% (*n* = 3). Horizontal—the time at which the cells were in hypoxic conditions. Vertical—the relative amount of mRNA, measured by the 2^-ΔΔCT^ method; box plots with whiskers at min to the max are presented. * The asterisk shows significant differences between the control and the 5% HX and 1% HX groups at *p* < 0.05 (unpaired nonparametric Mann–Whitney test).

To determine whether hypoxia is a sufficient factor to activate the Notch signaling pathway and *Bmp2/Runx2* factors, we took CMC from the healthy myocardium of sham-operated rats and induced hypoxia in vitro. Two different oxygen concentrations of 1% and 5% in the incubator and two time points of 8 and 24 h, during which the cells were under conditions of hypoxic stress, were chosen. We showed (Figure 8) that under conditions of hypoxia, the Notch signaling pathway components and *Bmp2/Runx2* are activated in cells compared to CMCs under normoxia conditions. *Hif-1α* and *Vegfa* were used as a control of hypoxia.

### 3.9. Exogenous Activation of the Notch Signaling Pathway in Cardiac Mesenchymal Cells Dose-Dependently Activates Runx2

**Figure 9:**
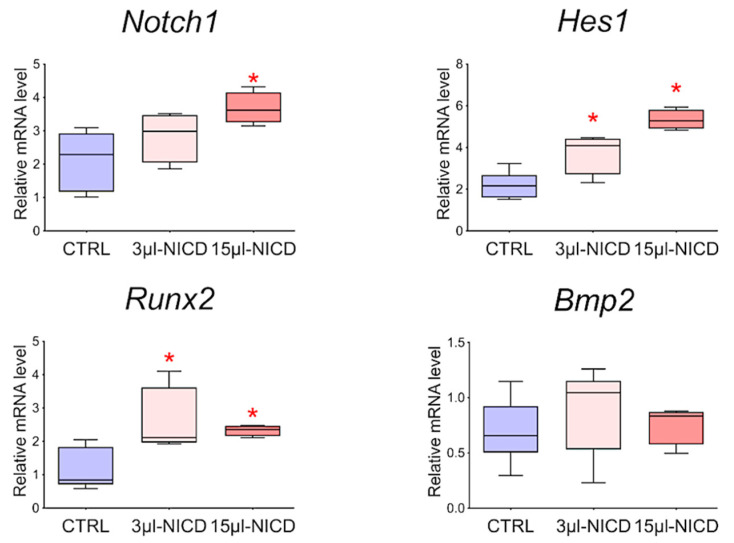
The dynamics of the expression of *Bmp2/Runx2* factors and Notch signaling pathways in cardiac mesenchymal cells obtained from healthy myocardium of sham-operated rats by quantitative PCR analysis. CTRL—healthy cells as a negative control (*n* = 3); 3 μL-NICD—healthy cells that had 3 μL of the virus with NICD added (*n* = 3); 15 μL-NICD—healthy cells that had 15 μL of the virus with NICD added (*n* = 3). Vertical—the relative amount of mRNA, measured by the 2^−ΔΔCT^ method; box plots with whiskers at min to the max are presented. * The asterisk shows significant differences between CTRL and the 3 μL-NICD and 15 μL-NICD groups at *p* < 0.05 (unpaired nonparametric Mann–Whitney test).

To reveal the relationship between the Notch signaling pathway and *Bmp2* and *Runx2* factors, we took CMCs from the healthy myocardium of sham-operated rats and transduced them with a lentiviral vector carrying an NICD insertion to activate the Notch signaling pathway. Viral particles were added to the culture at two different concentrations of 3 and 15 μL. Cells were cultured with the virus for 24 h. We demonstrated (Figure 9) that activation of the Notch signaling pathway led to dose-dependent activation of *Runx2*. On the contrary, the *Bmp2* did not respond to the activation of Notch.

## 4. Discussion

Myocardial infarction is a common acute disease that impairs heart functionality. Molecular and cellular mechanisms of cardiac early remodeling and recovery of postinfarction myocardium remain not fully understood. In this study, we induced myocardial infarction in rats to study early transcriptomic events occurring 8 and 24 h after surgical procedures. We showed a change in the expression pattern of Notch-associated genes and *Bmp2/Runx2* in postinfarction tissues using RNA sequencing and RT-PCR. We obtained primary CMC cultures from the postinfarction myocardium, which retained part of the activation stimulus and had a pronounced proliferative potential. Hypoxia in vitro also led healthy CMCs to upregulate the expression of Notch-associated genes and *Bmp2/Runx2*. Exogenous activation of the Notch signaling pathway resulted in a dose-dependent activation of the *Runx2* transcription factor but did not affect the activity of the *Bmp2* factor.

Some studies have shown that foci of proliferative activity are formed in the peri-infarction area in response to injury [2,22]. The effect of hypoxemia on myocardial recovery after a lesion has also been noted in in vivo studies in mice [23]. This study aimed to study early transcriptional events in cardiac tissue after myocardial infarction and to explore the cell population of cardiac mesenchymal cells, which can be isolated from myocardial tissue in order to analyze the molecular mechanisms of activation of the regenerative potential on these cells in vitro. 

The results of RNA sequencing during induction of myocardial infarction showed significant changes in the gene expression pattern in postinfarction tissue. Myriad signaling pathways and processes associated with early cardiac remodeling, cell proliferation and migration, and immune response have been affected.

Gude et al. showed that expression of the *Notch1* receptor, the *Jagged1* ligand, and the *Hes1* target gene upregulated in interstitial cells and cardiomyocytes in the peri-infarct region [24]. Notch is a highly conserved signaling pathway involved in the embryonic development of most multicellular organisms, as well as in the regulation of tissue homeostasis, cell differentiation, and maintenance of the stem cell population in the postnatal period [25]. The role of Notch in myocardial recovery is still not fully understood [26]. 

We observed that components of the Notch signaling pathway are activated in postinfarction tissue. Overexpressed genes included targets from non-canonical signaling, such as *Myc*, *Ccnd1*, and *Runx2* [14,15]. The *Runx2* transcription factor has been noted in the development of the cardiovascular system [27]. BMP signaling is also involved in development [28,29], and according to our data, it is activated during myocardial infarction, in particular, *Bmp2*. It is known to play a critical role in the development of the heart, induction of differentiation of cardiac progenitor cells into cardiomyocytes, and to stimulate their contraction [30,31,32,33]. It can act with a key upregulation role and modulate the Notch signaling pathway [34], and be involved in early remodeling processes [35]. Expression of *Bmp2* has been observed in both cardiomyocytes and interstitial fibroblasts in myocardial infarction [36,37]; however, the activation mechanism also remains unknown [38]. We hypothesize that the Notch signaling pathway, together with the key factors *Bmp2* and *Runx2*, may play an important role in early myocardial events in response to injury.

In order to confirm that, we obtained a fraction of activated cardiac mesenchymal cells from the post-infarction area of the myocardium with promoted properties. The discovery and experimental use of cardiac mesenchymal cells have become a new focus in cardiovascular regenerative medicine [39]. Recently, clinical trials have been conducted using one type of these cells and their products in therapy, and mainly to improve the function of the heart with a single ventricle in patients with hypoplastic left heart syndrome [40,41,42]. We obtained cells 24 h after the induction of myocardial infarction, and during cultivation, they were passaged only three times to preserve their properties and perform functional tests. Proliferative activity was assessed in real-time using the xCELLigence system. We found that post-infarction cardiac mesenchymal cells have a more pronounced proliferative potential than CMCs obtained from a healthy heart. This is consistent with the data which we obtained on tissues with the analysis of the affected processes. In turn, we performed RNA sequencing of primary cultures of CMCs and found that postinfarction CMCs partially retain their transcriptional profile and reflect early events in the affected myocardium.

For comparison, we obtained an activated CMC fraction 8 h after induction of myocardial infarction. The subsequent analysis of the evaluation of the expression of the Notch signaling pathway components and putative early remodeling genes using real-time PCR showed that the Notch target gene *Hes1* and *Bmp2/Runx2* factors are activated. Moreover, the activation of the latter was expressed precisely at an earlier time point.

Hypoxia has been described as a factor modulating Notch [43], and whether in vitro hypoxic stress on healthy CMCs can lead to the same activation of the Notch signaling pathway and early remodeling genes remains a question. We induced hypoxia in vitro by placing cardiac mesenchymal cells obtained from sham-operated rats in an oxygen-controlled incubator. CMCs experienced acute hypoxic stress at 1% or 5% oxygen for 24 h. We isolated the RNA and assessed gene expression. We found that in vitro hypoxia increased the expression of Notch signaling pathway components and *Bmp2/Runx2* genes.

In embryonic development, there is crosstalk between the Notch signaling pathway and *Bmp2*, as with *Runx2*, but whether Notch can act as an activator of putative early remodeling factors in healthy CMCs remains unclear. We transduced healthy CMCs obtained from sham-operated rats, a lentiviral vector in a low and high dose containing the insertion of the Notch intracellular domain 1 (NICD1). We showed that *Runx2* is dose-dependently activated in response to increased Notch expression. *Bmp2* did not react in any way to the introduction of the vector, which, in turn, may indicate its superior position in the regulation of the gene network, but we plan to investigate this interaction in the future.

Despite these results, our work may have the following limitations. Several studies have shown [44,45,46] the probable effect of epicardium-derived cells on neovascularization and cardiomyogenesis by reactivating a fetal gene program in response to myocardial infarction. In our methodology, we removed the epicardium prior to isolation of RNA and primary cell cultures, but there is a possibility that the influence of the activated epicardium may have affected the myocardium, and therefore further studies are required to identify possible effects. In addition, we would like to note that under in vitro cultivation conditions, we inevitably encounter a change in the properties of cultured cells, which in turn can affect the ability of mesenchymal cells to proliferate and change their proangiogenic properties [47]. In this work, we showed that under the same conditions, postinfarction CMCs had more pronounced proliferative abilities than CMCs that were obtained from healthy animals, but whether this will affect myocardial recovery in vivo remains a question for further studies.

## 5. Conclusions

Recovery of the contractile function of the heart and the regeneration of the myocardium after ischemic injury are contemporary issues in regenerative medicine and cell biology. Traditional treatments such as drug therapy and revascularization only cropped symptoms but do not contribute to full recovery from developing heart failure.

Thus, the results of this study showed that acute hypoxic stress could cause short-term activation of the embryonic signaling pathways Notch and Bmp in cardiac mesenchymal cells, and this interaction is closely related to the processes of early myocardial remodeling after a heart attack. The ability to correctly modulate and control the corresponding signals in the heart can help increase the regenerative capacity of the myocardium before the formation of fibrotic conditions.

## Data Availability

Data is contained within the article and supplementary files. Raw and processed RNA sequencing data were uploaded to the NCBI website as specified in the rules for authors. They are available at the following link: https://www.ncbi.nlm.nih.gov/geo/query/acc.cgi?acc=GSE201888 (accessed on 2 May 2022).

## Supplementary Materials

The following supporting information can be downloaded at: https://www.mdpi.com/article/10.3390/biomedicines10061283/s1. Figure S1: A. Dysregulation of biological pathways (excluding metabolic pathways) in post-infarct tissue samples identified by IPA analysis. The histogram represents the dysregulated canonical signaling pathways in a stacked bar chart by Fisher’s Exact Test p-value (adj. p-value > 0.05, z-score > 1). B. Dysregulated gene networks involved in the growth and development of tissues and cells, which were generated using the IPA software. Red means increased measurements, and green means decreased amounts. The brightness of the color means the more extreme of the transcripts in this dataset; Figure S2: Upregulated bio functions involved in the proliferation and differentiation of the heart cells, which were identified by GSEA using the IPA software. Red means increased measurements, and green means decreased amounts. The brightness of the color means the more extreme of the transcripts in this dataset; Table S1:Top 50 DEGs in postinfarction rat myocardial tissues sorted by stat using DeSeq analysis; Table S2:Top Diseases and Bio Functions in postinfarction tissues by IPA Software; Table S3: Components of the Notch signaling pathway that are differentially expressed in postinfarction myocardial tissues 24 hours after surgery; Table S4: Full list of differentially expressed genes in post-infarction cardiac mesenchymal cells; Table S5: A complete list of expression genes in post-infarction cardiac mesenchymal cells, assorted by a statistical criterion (P-Value <0.05); Table S6: Components of the Notch signaling pathway that are differentially expressed in postinfarction CMC; Table S7: A complete list of signaling pathways with involved genes is shown in the table, extracted using the IPA program; Table S8: A complete list of diseases and biofunctions with involved genes is presented in the table obtained using the IPA program; Table S9: Probabilistic networks of dysregulated genes created with the IPA software.
