# Supplementary figures and images for: Mechanisms of Regenerative Potential Activation in Cardiac Mesenchymal Cells
Source: Biomedicines. 2022 May 31;10(6):1283. doi: 10.3390/biomedicines10061283 (PMC9220771; doi:10.3390/biomedicines10061283)

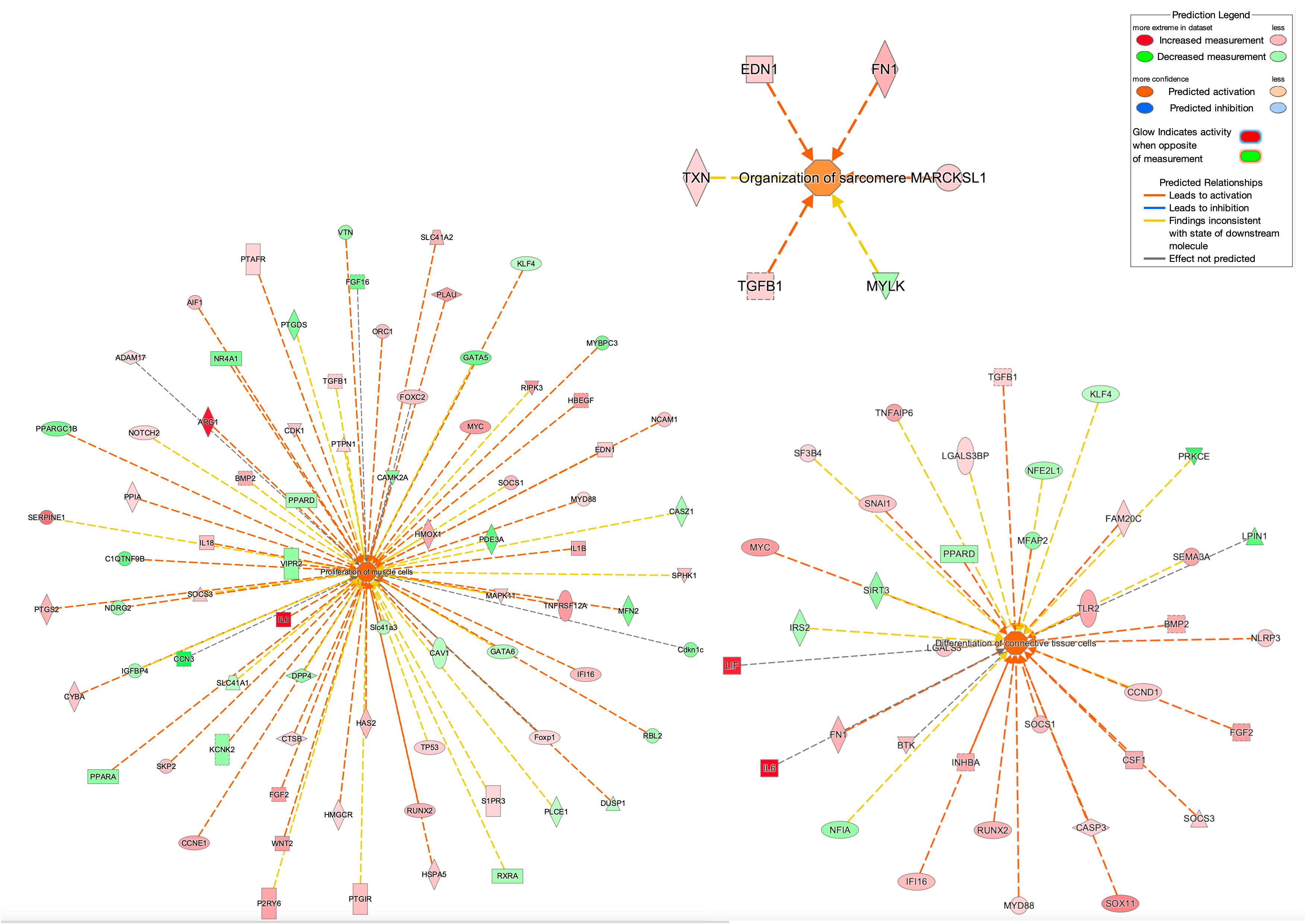

Supplement: Supplementary file 1 [file biomedicines-10-01283-s001.zip › Figure S2.tif]

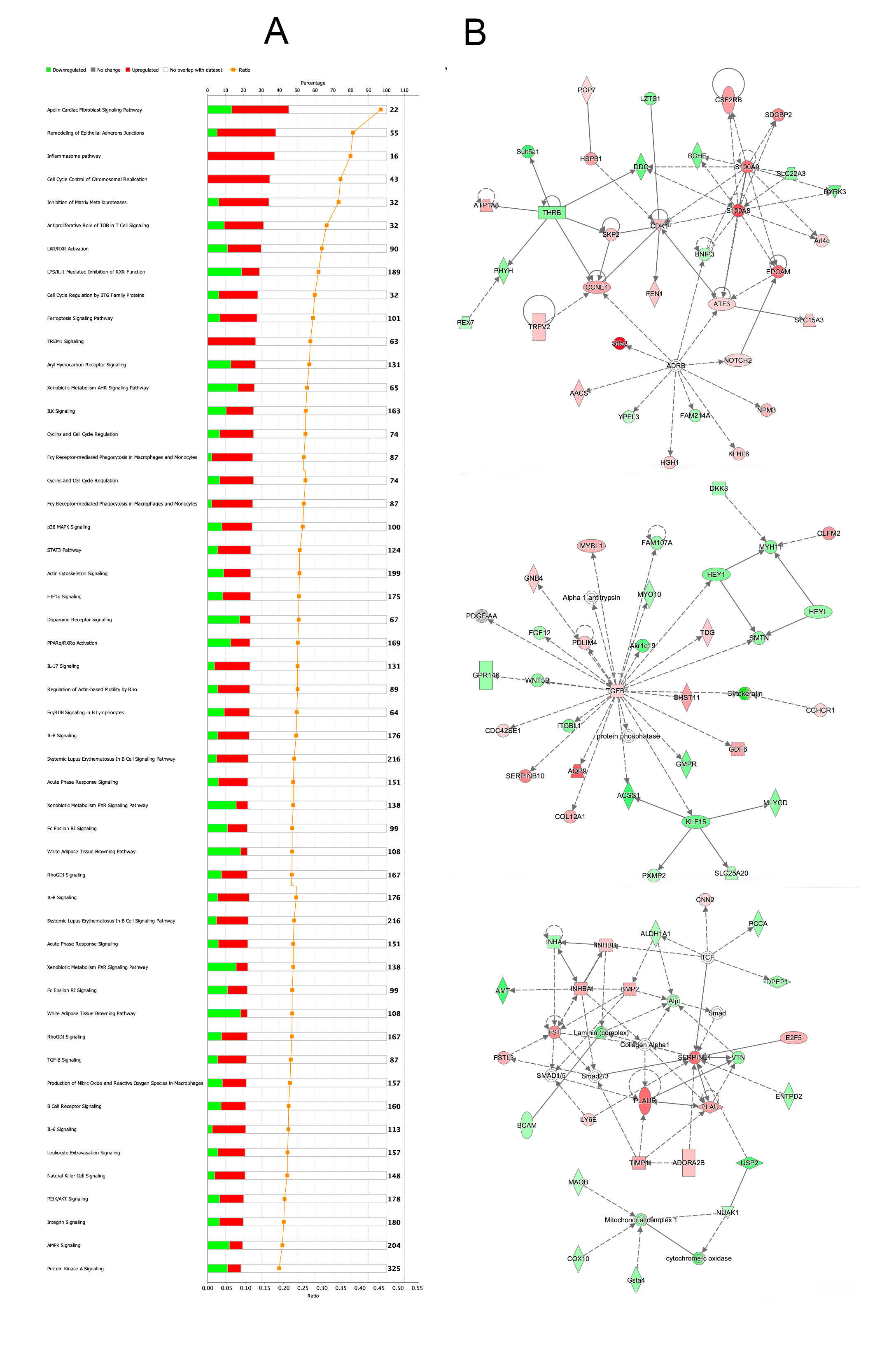

Supplement: Supplementary file 1 [file biomedicines-10-01283-s001.zip › Figure S1AB .tif]
